# The Role of Endobronchial Biopsies in Evaluating Biologic Therapy Response in Severe Asthma

**DOI:** 10.3390/ijms26167692

**Published:** 2025-08-08

**Authors:** Agamemnon Bakakos, Dimitrios Ampazis, Andriana I. Papaioannou, Stelios Loukides, Petros Bakakos

**Affiliations:** 1 1st Department of Respiratory Medicine, National and Kapodistrian University of Athens, 11527 Athens, Greece; papaioannouandriana@gmail.com (A.I.P.); petros44@hotmail.com (P.B.); 2 Respiratory Department, Cavan & Monaghan Hospital & Chronic Disease Management Hub, HSE & RCSI University of Medicine, H12 Y7W1 Cavan, Ireland; dim_ampazis@yahoo.gr; 3 Royal College of Surgeons in Ireland, University of Medicine & Health Sciences, D02 YN77 Dublin 2, Ireland; 4 2nd Department of Respiratory Medicine, National and Kapodistrian University of Athens, 11527 Athens, Greece; loukstel@med.uoa.gr

**Keywords:** asthma, severe asthma, biologics, biopsies

## Abstract

Severe asthma imposes a significant burden on public health worldwide, mainly due to its morbidity and high cost. The management of severe asthma has dramatically changed in the past few years with the introduction of biologics. Zero exacerbations, zero systemic corticosteroids, better asthma control, and better lung function are the outcomes that the era of biologics has made attainable in a large proportion of severe asthmatics, ending up in a better quality of life. Still, even today, the changes at the tissue level that reflect these outcomes are not that clear. As a chronic inflammatory disease, asthma often involves airway remodeling in its severe forms; endobronchial biopsies may provide critical insights into these tissue-level changes before and after biologic treatment. However, bronchoscopy is an invasive tool for severe asthma, thus limiting its use in daily clinical practice. This review focuses on summarizing the changes that biologics exert in biopsies obtained from severe asthmatics under biological treatment, providing an opportunity to shed light on what really happens there where it is not easy to see, and especially on what does not happen in patients under biologics who fail to respond as expected. Moreover, the armamentarium of biomarkers used for making the proper choice in patients eligible for more than one biologic needs to be enriched. Biopsy-related markers could be an ideal adjunct to the current ones—blood eosinophils, FeNO, and IgE—to assist the clinician to choose the right biologic for the right patient with severe asthma to achieve disease remission.

## 1. Introduction

Asthma imposes a tremendous burden in public health worldwide by affecting more than 330 million people [1]. Despite the fact that what is defined as severe asthma accounts for less than 5–10% of the total asthmatics, the majority of asthma cost is attributed to this population [2]. Quite recently, the landscape in the management of severe asthma has dramatically changed due to the treatment modality of biologics, and this has been vividly reflected in the respective guidelines. Our arsenal so far involves six biologics—omalizumab, mepolizumab, benralizumab, reslizumab, dupilumab and tezepelumab—that have been approved for severe asthma and have been embedded in the guidelines, making severe asthma a completely different disease phenotype compared to what it was supposed to be 20 years ago [1]. The cornerstone for the choice of the most appropriate biologic is the complete phenotyping and endotyping of the severe asthmatic patient [3]. Taking into account that one patient with severe asthma may be eligible for more than one biologic, the process of such phenotyping—which should also include the presence of comorbidities—becomes more necessary and makes it imperative to gain a deep understanding not only of the pathophysiology of severe asthma but also of the mechanisms of action and the exact effects of biologics at the tissue level. Patients with severe asthma are those who are controlled only when treated with Global Initiative for Asthma (GINA) step 4 or 5 or remain uncontrolled despite this treatment [1,4]. For many asthmatics who receive treatment GINA step 4 or 5, the lack of disease control is attributed to a poor inhaler technique, low adherence to medication, and also the existence of comorbidities comprising the population of difficult-to-treat asthma [5]. Asthmatics who remain uncontrolled after taking care of these factors are eventually defined as suffering from severe asthma [6]. Frequent asthma exacerbations and daily use or frequent bursts of systemic corticosteroids are often encountered in severe asthmatics, and this is associated with adverse events, frequent physician visits, and hospital admissions [7]. The elimination or reduction of systemic corticosteroids has always been a priority target in severe asthma [8].

Severe asthma presents significant heterogeneity regarding age onset, clinical presentation, inflammatory mechanisms, and response to treatment [9]. In terms of inflammation, asthma is often characterized by eosinophilic airway inflammation. In T2 high asthma, type 2 inflammation with eosinophilic infiltration of the airways is typical; thus, blood eosinophil count and fractional exhaled nitric oxide (FeNO) are the most established biomarkers used in everyday clinical practice for the recognition of this phenotype [10]. In T2 low asthma, different inflammatory pathways are involved, which might include neutrophils or even a lack of inflammatory cells (paucigranulocytic asthma) [9]. The T2 high phenotype is by far the most frequent, accounting for 60–70% of severe asthma cases [11,12]. Biologics are a treatment option for T2 high severe asthma but also have the advantage of treating several comorbidities such as atopic dermatitis, chronic rhinosinusitis with nasal polyps, and chronic urticaria, thus targeting and improving more than one disease at the same time [13,14,15,16,17,18]. Severe asthma phenotyping is mainly based upon two biomarkers: peripheral blood eosinophils and FeNO. However, induced sputum has also been used. Nevertheless, underlying asthma treatment may reduce blood eosinophils, FeNO, and even affect sputum cell populations, as steroids reduce sputum eosinophils and may increase sputum neutrophils [19,20]. Additionally, induced sputum is a time-consuming semi-invasive procedure, requiring special equipment, which is challenging to perform, especially in severe asthmatics and definitely not repeatedly in order to assess changes due to treatment interventions. Moreover, airway remodeling, another aspect of asthma pathophysiology that has been shown not to be directly related to inflammation, cannot be evaluated by blood nor by sputum [21]. It requires endobronchial biopsies that possess even more difficulties than induced sputum in order to be obtained in severe asthmatics.

Our understanding of changes occurring in severe asthma might benefit from examining them at the tissue level; however, the difficulty in obtaining biopsies from patients with severe asthma through bronchoscopy—especially after treatment with a biologic—hinders the recognition of the precise changes that translate into the final treatment outcome. Accordingly, only few studies in asthma and even less in severe asthma have assessed the effect of a biologic treatment by evaluating bronchial biopsies.

The aim of this review is to present evidence from studies that performed bronchial sampling via biopsy to examine the immunologic and structural changes associated with biologic treatment in severe asthma. By focusing on histopathological findings, cytokine expression, and airway remodeling profiles, this review aims to offer mechanistic insight into the actions of biologics and to highlight the potential role of bronchial biopsies as a tool for individualized clinical decision-making in severe asthma care.

## 2. Omalizumab

The mechanism of action of omalizumab is binding free IgE and hindering its binding to the high affinity receptor on basophils and mast cells, thus reducing IgE levels and downregulating the receptor FcεRI [22]. Omalizumab is indicated in moderate-severe atopic asthma with at least one positive skin prick test or RAST to a perennial allergen and the dosage is calculated according to body weight and initial level of total IgE [23].

In a prospective, randomized study, 45 individuals with mild to moderate persistent asthma who exhibited elevated sputum eosinophils (≥2%) received omalizumab (*n* = 22) or placebo (*n* = 23) for 16 weeks. Bronchial biopsies were obtained at baseline and following treatment, and the study demonstrated that omalizumab led to a marked reduction in multiple inflammatory cell populations, including eosinophils, IgE-expressing cells, T-cell subsets (CD3^+^, CD4^+^, CD8^+^), B-lymphocytes, and IL-4-positive cells, indicating a broad suppression of allergic inflammation at the tissue level. However, these histological improvements did not translate into changes in airway hyperresponsiveness, as determined by methacholine challenge testing. This discrepancy between immunopathological improvement and bronchial reactivity highlights the complexity of severe asthma and suggests that airway remodeling or non-eosinophilic mechanisms may persist despite reductions in eosinophilic inflammation [24].

A double-blind, randomized, placebo-controlled trial included 25 participants, all of whom underwent an inhaled allergen challenge, followed by bronchoscopy 24 h later to assess acute mucosal changes. After being randomized to receive either omalizumab or placebo, participants were treated for 12 weeks, at which point repeat bronchoscopies were performed. Additional measurements, including induced sputum analysis and methacholine bronchial challenge tests, were conducted at baseline, week 8, and week 12. The omalizumab-treated group demonstrated significant reductions in airway eosinophils, as well as CD4^+^ T cells and cells bearing high-affinity IgE receptors (FcεRI). These findings indicate that omalizumab exerts a measurable anti-inflammatory effect at the tissue level, even within a relatively short treatment period [25]. However, these immunological improvements did not coincide with changes in airway hyperresponsiveness [25].

In a prospective, non-randomized study including 11 patients with severe persistent allergic asthma, all of whom were followed over a 12-month treatment period, the long-term impact of omalizumab on airway structural changes was evaluated. Bronchial biopsies were obtained before and after treatment. Following one year of omalizumab therapy, patients exhibited a notable reduction in both RBM thickness and eosinophilic inflammation, suggesting that the drug may help attenuate chronic remodeling processes within the airways [26]. Interestingly, the observed reductions in RBM and eosinophil counts did not correlate with one another, nor did they show a clear association with clinical parameters, such as lung function or symptom control. Nevertheless, the structural improvements were considered indicative of a potential disease-modifying effect, supporting omalizumab’s role in reversing or stabilizing airway remodeling in severe allergic asthma [26].

A prospective, non-randomized study explored the potential of galectin-3 as a biomarker for airway remodeling response in patients with severe persistent atopic asthma undergoing treatment with omalizumab. This study involved eight patients, the majority of whom were current or former smokers, with reduced lung function (mean FEV_1_ ~56%) and elevated serum IgE levels at baseline. All participants underwent bronchial biopsies of the right middle lobe before starting therapy and again after 12 months of treatment. Histological analysis was primarily directed at changes in the reticular basement membrane, while proteomic profiling of biopsy specimens was conducted using multidimensional protein identification technology (MudPIT). Post-treatment samples showed a notable decrease in bronchial smooth muscle-associated proteins, including myosin and actin, suggesting a biologically relevant effect of omalizumab on the contractile elements involved in airway remodeling. However, markers of the extracellular matrix, such as vimentin and periostin, remained unchanged [27]. A key finding was the selective presence of galectin-3—a protein with known IgE-binding properties—in patients who responded clinically to omalizumab. Galectin-3 was absent in non-responders, but detectable in both biopsy tissue and biological fluids (serum and urine) of responders, supporting its potential use as a non-invasive biomarker for identifying patients likely to benefit from anti-IgE therapy [27]. These results point to a mechanistic link between omalizumab and airway remodeling reversal, potentially mediated by the modulation of smooth muscle protein expression and disruption of the IgE-FcεRI axis.

A prospective, randomized clinical trial explored the mucosal effects of omalizumab in patients with symptomatic non-atopic asthma, a population less commonly studied in the context of anti-IgE therapy. A total of 18 patients were randomized in equal numbers to receive either omalizumab or placebo over a 20-week period. Bronchial biopsies were collected prior to treatment initiation and again at 12–14 weeks, with tissue analyzed for the presence of IgE-positive cells, mast cells, plasma cells, B-lymphocytes, eosinophils, and plasmablasts. After the second biopsy, all participants underwent a supervised reduction in baseline asthma therapy, with pulmonary function assessed at week 20 to observe any effects from therapeutic destabilization [28]. The most significant histological finding was a notable decline in IgE-expressing cells within the airway mucosa of the omalizumab-treated group. No substantial differences were noted in the other inflammatory cell populations. Functionally, individuals receiving omalizumab showed preserved or improved FEV_1_ following treatment tapering, while those on placebo exhibited a decline in lung function during the same period [28]. These results suggest that even in the absence of atopy, targeting IgE may yield clinical benefits, potentially stabilizing lung function despite minimal changes in broader airway inflammation.

A prospective, non-randomized study enrolled 23 individuals with unstable severe allergic asthma, of whom 14 exhibited clinical improvement following anti-IgE therapy. Notably, 12 of these 14 responders were classified within the type 2 high inflammatory endotype, characterized by elevated tissue expression of IL-33, IL-25, and TSLP detected in pre-treatment bronchial biopsies. Cytokine levels were analyzed using Western blot techniques for mRNA expression, with the biomarkers predominantly localized to the epithelial layer, endothelium, and subepithelial inflammatory infiltrates. Among the responders, post-treatment biopsy samples showed substantial reductions in IL-33, IL-25, and TSLP, along with decreased levels of IL-13 and IL-9, albeit to a lesser degree. These immunological shifts correlated with fewer exacerbations, improved asthma control, and better lung function outcomes [29]. Those benefiting most from omalizumab were typically female, non-smokers, with high FeNO, eosinophilic airway inflammation, and poorly controlled asthma at baseline. In contrast, partial responders more often presented with mixed eosinophilic–neutrophilic inflammation, concomitant chronic rhinosinusitis, and required adjunctive macrolide therapy [29]. These findings underscore the potential of IL-33, IL-25, and TSLP as biomarkers of treatment responsiveness, supporting their integration into biopsy-based endotyping strategies to inform personalized biologic selection in severe allergic asthma.

A clinically instructive case of a 65-year-old woman with severe allergic and eosinophilic asthma, who initially responded well to omalizumab but developed an atopic hypersensitivity reaction after the fourth dose, prompted a temporary discontinuation and the implementation of a desensitization protocol. To inform further therapeutic direction following desensitization, the care team performed bronchoscopy with endobronchial tissue sampling. Histological evaluation revealed high levels of IgE expression within the airway epithelium, accompanied by low-intensity IL-5 staining in the submucosal layer. This IgE-dominant inflammatory profile, with only modest involvement of IL-5-driven pathways, supported the decision to reintroduce omalizumab rather than transition to an anti-IL-5 biologic [30]. Following re-initiation, the patient again experienced clinically significant improvements, including enhanced FEV_1_ and better asthma symptom control. This case demonstrates the value of bronchoscopic immunophenotyping in guiding biologic selection in severe asthma, particularly when clinical presentation overlaps with multiple inflammatory endotypes [30].

A prospective, non-randomized study examined the structural impact of omalizumab on the airway wall in 13 patients with severe allergic asthma. All patients were monitored over a 12-month treatment course and tissue samples were obtained from the bronchial mucosa and bronchoalveolar lavage (BAL) fluid during bronchoscopy at baseline and one year post-treatment. After one year of omalizumab therapy, patients demonstrated a significant reduction in basal lamina thickness and fibronectin deposition, suggesting a partial reversal of remodeling processes associated with chronic asthma. In contrast, collagen levels remained unchanged, indicating a selective structural effect limited to specific matrix proteins and epithelial features. The decrease in fibronectin was positively correlated with improvements in asthma control and quality of life scores. Furthermore, many patients were able to reduce their reliance on oral corticosteroids, and a trend toward reduced eosinophilic inflammation was observed across blood, BAL, and tissue compartments [31]. These findings support the notion that omalizumab may exert modifying effects on airway architecture, particularly through its impact on fibronectin and epithelial remodeling, thereby enhancing its value beyond inflammation control in severe allergic asthma.

## 3. Mepolizumab

Mepolizumab is a mAb which inhibits interleukin-5 (IL-5) and hinders the differentiation, maturation, migration and finally the survival of eosinophils in lung tissue [32]. The well-known randomized control trials (RCTs) which led to the approval of mepolizumab for T2 high severe asthma all shared a common feature. The higher the number of blood eosinophils of the patient, the higher the efficacy of treatment with the biologic [33,34]. From the very beginning, blood eosinophils were used as a surrogate marker of lung eosinophilia, mainly because obtaining a peripheral blood count is a much easier and safer procedure than the measurement of lung or sputum eosinophils [35]. Nevertheless, it is interesting to address the direct effects of mepolizumab treatment at the site of interest, which is no other than the lung tissue.

Although mepolizumab can greatly reduce the number of blood and lung eosinophils, those that remain after the inhibition of IL-5 could be totally functional. This was further evaluated in a trial where patients with mild allergic asthma underwent segmental bronchoprovocation with allergen before and one month after having received a single 750 mg mepolizumab dose. Baseline bronchoscopy was performed and two days after the allergen challenge, patients underwent a second bronchoscopy. In both examinations, researchers obtained bronchoalveolar lavage (BAL) and endobronchial biopsies, but in the latter one, biopsies were both taken from segments which were antigen-naïve and from the high-dose segments. A peripheral blood specimen to measure blood eosinophils was also collected [36].

As expected, the allergen challenge in the pre-mepolizumab group resulted in an increased number of blood eosinophils and eosinophils in BAL and increased eosinophil peroxidase deposition in the bronchial mucosa. Mepolizumab managed to block the eosinophilic response after allergen challenge, with blood eosinophils and IL-3 receptor expression on them remaining low, showing its effect in altering their activation status. However, airway eosinophilia and deposition of eosinophilic peroxidase, although substantially reduced, were not completely abolished and the remaining airway eosinophils collected from the BAL were still able to express IL-5 and IL-3 receptors as well as cytokine receptors, despite treatment with mepolizumab. This finding could possibly explain why asthmatics under anti-IL-5 treatment can still experience an exacerbation despite the low number of circulating eosinophils [36].

One of the most comprehensive studies on the effects of mepolizumab on the airways and the structural changes it leads to is the MESILICO (Efficacy of Mepolizumab in patients with latE-onset Severe eosInophiLic asthma and fIxed obstruCtiOn) study, which is a multicenter study conducted in Greece. In this study, 41 severe asthmatics with T2 high eosinophilic asthma were recruited and performed baseline and 12 months post-mepolizumab treatment bronchoscopies, with obtainment of endobronchial biopsies. Out of the 41 patients, 34 had both pre- and post-treatment endobronchial biopsies. A significant reduction in sub-basement membrane thickness, airway smooth muscle (ASM) area, airway smooth muscle layer thickness, extent of epithelial damage, and number of tissue eosinophils was observed, with a *p*-value < 0.001 [37]. All of the above findings in tissue are hallmarks of airway remodeling in asthma, which in turn occurs due to chronic inflammation and in the end leads to fixed obstruction [38]. These critical findings suggest that biologic treatment in severe asthma could have an impact in reversing airway remodeling, which was otherwise considered to be non-reversible. Finally, the margin of thickness reduction in airway smooth muscle cells was directly correlated to the reduction in submucosal eosinophils, suggesting a direct mechanism of action underscoring the importance of blocking IL-5 with mepolizumab not only in terms of symptom control and exacerbation reduction but also in a “disease-modifying” way by reducing the extent of airway remodeling [37].

One of the first double-blind placebo-controlled trials for mepolizumab recruited 24 mild asthmatics and randomized them to either anti-IL-5 treatment or placebo in an early effort to evaluate its effect by obtaining endobronchial biopsies at baseline and three months post-treatment. Administration of mepolizumab managed to significantly reduce the number of eosinophils infiltrating the bronchial mucosa, reduced TGF-β expression, and also reduced the level of extracellular matrix proteins (such as tenascine) in the reticular basement membrane. This was the first evidence that mepolizumab can mitigate eosinophil-driven airway remodeling [39].

The reversal of airway remodeling was further validated in a single center French study, which was reported as an abstract in the American Thoracic Society (ATS) congress in 2024. A total of 37 severe asthmatics who were candidates for mepolizumab treatment were enrolled and bronchoscopy along with obtainment of BAL and endobronchial biopsies was performed before initiation of treatment and after 6 and 12 months, respectively. Almost half (23 out of 37) of patients had paired baseline and 12-month biopsies and in the latter biopsy, there was a significant reduction in smooth muscle cell area and sub-basement membrane thickness compared to baseline findings. Furthermore, eosinophilic infiltration was already mitigated after 6 months, and this effect persisted in the 1-year biopsy specimens. Concerning BAL findings, eosinophils were reduced to almost zero in most patients and the levels of several T2 inflammatory biomarkers, such as CCL11, CCL24, and tenascine, were significantly reduced as well [40].

## 4. Benralizumab

Benralizumab is another mAb targeting the IL-5 pathway, with a different mechanism of action. Instead of blocking IL-5, it blocks IL-5R (which is expressed at the surface of eosinophils and basophils) and exerts a unique antibody dependent cell-mediated cytotoxicity (ADCC) by natural killer (NK) cells, inducing apoptosis of eosinophils. This attribute is due to the lack of fucose (afucosulation), which in turn increases its affinity to CD16a. Not only does benralizumab hinder the IL-5 pathway but it also leads to a direct and fast reduction in eosinophil numbers in lung tissue and in peripheral blood [41]. The haste of its effect is reflected in this substudy of the Oxford Airways Study, in which benralizumab was compared to mepolizumab and prednisolone. Benralizumab expressed a profile which is similar to that of prednisolone, with blood eosinophil levels being largely depleted after just 24 h of administration. Although long term efficacy of mAbs is not based on how quickly they reduce eosinophil levels, the faster onset of action for benralizumab makes it a viable option in the treatment of an acute exacerbation with the benefits that non-corticosteroid treatment brings [42]. Another recent study has also demonstrated in a larger population with a T2 high acute exacerbation of either asthma or COPD the benefits of benralizumab treatment in an acute setting, compared to the standard of care use of prednisolone [43].

Its efficacy in reducing eosinophils in both lung tissue and peripheral blood has been demonstrated for more than a decade, before it received approval for severe eosinophilic asthma. Back in 2013, a phase I double-blind placebo-controlled study randomized 13 patients to a single intravenous dose of benralizumab or placebo and 14 patients to either placebo or a subcutaneous dose of benralizumab with a 28-day interval between each injection (doses were 100 mg or 200 mg and randomization rate was 1:1:1). Endobronchial biopsies were collected before the induction of treatment and after 3 months for the subcutaneous infusion cohort. This cohort expressed a combined median reduction of almost 96% in airway eosinophils and a 90% decrease in sputum eosinophils versus placebo after 3 months of follow-up. Additionally, eosinophils were completely depleted in peripheral blood, with a remarkable 100% reduction. It should be noted that the subcutaneous cohort results were by far superior compared to those of the intravenous infusion group of asthmatic patients. It is also important to underscore that blood eosinophils were undetectable after just 1 week of treatment with benralizumab [44].

The role of ASM cells in severe asthma and especially in airway remodeling has started gaining attention and the potency of benralizumab in reducing ASM mass has not been elucidated. In an effort to clarify its effects, researchers used the same biopsies that were obtained in the aforementioned trial of Laviolette et al. [44] and evaluated 15 patients from the treatment arm and 10 from the placebo arm. A statistically significant decrease in ASM mass was reported in the benralizumab group comparing baseline and post-treatment biopsies, with a mean reduction of 29% (*p*-value = 0.021); however, when comparing the post-treatment biopsies of the benralizumab and the placebo groups, the difference was insignificant (*p*-value = 0.475). Even though ASM cells do not express IL-5R and therefore the effects of benralizumab are most likely indirect, the small sample size and short follow-up duration of just 3 months limit the generalizability of these findings [45].

A more recent study recruited 46 patients with mild allergic asthma in an effort to determine whether administration of benralizumab in a dose of 30 mg every 4 weeks for 3 months could attenuate the allergen induced late asthma response (LAR). Patients were randomized in a 1:1 ratio to either treatment or placebo. Endobronchial biopsies were taken before the first dose and one month after the third dose of benralizumab (90 days after the first dose). Patients in the benralizumab arm showed a far more reduced eosinophilic proliferation compared to the placebo group 7 h after the allergen challenge, and especially in bronchial submucosa, the mean eosinophilic count was significantly greater (*p*-value = 0.005) in the placebo group 24 h after the allergen challenge in the 12th week of follow-up. The same results were noted also for eosinophils in specimens of sputum, blood, and bone marrow. Although the aim of reducing the allergen-induced bronchoconstriction by depleting eosinophils was not achieved, hinting that eosinophils are not the sole mediator of LAR in mild allergic asthma, this study indirectly managed to further validate the near complete depletion of airway and sputum eosinophils in asthmatic patients under treatment with benralizumab [46].

Currently, there is a large phase 4 real-world study ongoing, which is evaluating the results of treatment with benralizumab on structural and lung function changes in severe asthmatics. It is estimated to be completed by the end of 2026. In this study, researchers aim to explore the effects of benralizumab on airway tissue eosinophils, assess airway remodeling features through endobronchial biopsies, and finally correlate their findings with changes observed in lung function tests. It will be pivotal to prove its efficacy in altering the remodeling process in severe asthma [47].

Finally, another study focusing on remodeling currently in the recruitment phase is about to enroll a total of 60 patients, with 20 of them being severe asthmatics, 20 non-severe asthmatics, and 20 non-asthmatics. Endobronchial biopsies will be obtained from all patients and airway remodeling parameters will be evaluated in vitro in the presence and absence of benralizumab, respectively, for all patients [48].

## 5. Dupilumab

Dupilumab is a biologic agent which targets both IL-4 and IL-13 by binding to the IL-4R α subunit. There are two IL-4R forms, type I and type II. The first one can only bind to IL-4, while the second one can bind to both IL-4 and IL-13. Dupilumab manages to inhibit signaling from both types of the IL-4R, thus hindering the effects of IL-4 and IL-13. IL-4 is produced by Th2 cells, eosinophils, basophils, mast cells, ILC2, and macrophages and plays a significant role in the inflammatory cascade of T2 high response. IL-13 has been implicated in the thickening process of the basement membrane by inducing collagen deposition and in the hypertrophy of the smooth muscle cells.

A key difference between dupilumab and the mAbs targeting IL-5 is that dupilumab can cause transient eosinophilia in a fair percentage of patients, from 4% up to 25%. Although clinically significant implications are scarce and patients’ response is not determined by it, sometimes blood eosinophil levels increase dramatically, which in turn means that they need to receive treatment to reduce their number [49].

Until now, there is no widely available data for bronchoscopic findings in patients under dupilumab. A phase IIa exploratory trial under the name EXPEDITION was the first trial to evaluate dupilumab’s effects in the airways by obtaining baseline bronchial biopsies. A total of 42 patients were randomized to either dupilumab or placebo in a 1:1 ratio and follow-up consisted of 12 weeks of treatment, after which bronchial biopsies were obtained again. Compared to baseline measurements, eosinophil and mast cell levels were not reduced in bronchial submucosa. On the other hand, the mucin-stained area in the submucosa significantly decreased in patients under dupilumab along with FeNO levels, indicating that dupilumab affects and restrains T2 inflammation without affecting eosinophil and mast cell levels [50].

In another small series of severe asthmatic patients (*n* = 10) treated with dupilumab for at least 6 months, researchers performed bronchoscopy and obtained BAL and bronchial biopsies at baseline and after 6 months. Eosinophils were abolished in the BAL fluid after 6 months (0–1%); however, there was a persistent presence of eosinophils in the bronchial mucosa, despite patients’ improvement in terms of exacerbation reduction, while all of them managed to discontinue corticosteroid treatment. This evidence highlights once again the different mechanism of action through which dupilumab achieves suppression of T2 inflammation that does not necessarily involve complete elimination of tissue eosinophils [51].

## 6. Tezepelumab

The “newer kid on the block” is tezepelumab. Tezepelumab is directed against TSLP, an alarmin mainly released by epithelial cells. Its mechanism of action is mediated through binding to TSLP and prevention of interaction with the TSLP receptor [52]. Tezepelumab, apart from reducing all T2-high biomarkers, and thus blood eosinophils, FeNO, and IgE, demonstrated efficacy in severe asthma with not remarkably high T2 biomarkers (T2 low asthma), but still to a lesser extent compared to T2-high asthma [53,54].

The phase 2, multicenter CASCADE trial assessed the impact of tezepelumab on airway inflammation, remodeling, and hyperresponsiveness in patients with moderate to severe uncontrolled asthma. This study employed a randomized, double-blind, placebo-controlled design, enrolling 250 patients, with 116 ultimately randomized—59 to tezepelumab and 57 to placebo. A majority of these completed the study’s primary endpoint at 28 weeks, with some follow-up extended to 52 weeks due to COVID-19-related delays [55].

All participants underwent bronchoscopic tissue sampling at baseline and post-treatment. Histological analysis focused on submucosal inflammatory cell populations, including eosinophils, neutrophils, CD3^+^ and CD4^+^ T cells, and mast cell subtypes according to the protease they release upon stimulation (tryptase^+^ and chymase^+^). Additional secondary endpoints included reticular basement membrane (RBM) thickness, epithelial integrity (assessed by characterizing the epithelium as either denuded, damaged, or intact), and airway hyperresponsiveness to mannitol challenge. Notably, this study was planned to include further findings relevant to airway submucosal inflammatory cells and additional RNA assay from bronchial brushings sampling. This, however, was not ultimately achieved due to technical inaccuracies of the assay [55].

This study’s most prominent finding was a marked 89% reduction in submucosal eosinophils in the tezepelumab group, compared with a 25% reduction in the placebo group. This selective eosinophil depletion was evident irrespective of the initial high Th2 or low Th2 status. However, the bronchial eosinophilic change was not accompanied by substantial changes in other inflammatory cell subsets, suggesting a targeted anti-type 2 inflammatory effect. Interestingly, there was no decrease in the levels of neutrophils or lymphocytes, a finding supportive of tezepelumab’s safety profile regarding immunosuppression and potential increase in infective episodes [55].

Notably, the airway eosinophil reduction in the Th2-low subgroup occurred despite conventional biomarkers of eosinophilic and atopic inflammation being low (blood eosinophils < 150 cells per μL, FeNO < 25 ppb, serum IgE, IL-5, and IL-13 lower-than-median serum concentrations). This is indicative of tezepelumab’s potential to either affect Th2 pathways or non-Th2-mediated mechanisms and improve asthma phenotypes that currently do not meet the criteria of atopic/eosinophilic features as per the conventional thresholds of biomarkers [55].

A further finding, indirectly suggestive of some improvement in the remodeling features, refers to the increase in airway lumen as per the cross-sectional CT imaging findings, suggesting possible structural changes in the treatment group. It is worth noting, though, that other indices—such as epithelial morphology, reticular basement membrane thickness, or further CT imaging criteria—could not confidently suggest remodeling improvement in the tezepelumab group [55].

These results affirm tezepelumab’s role as a disease-modifying agent, particularly in patients with eosinophilic asthma, by demonstrating its capacity to reduce airway tissue eosinophilia and potentially attenuate underlying airway remodeling and hyperresponsiveness.

Similar findings had been identified earlier by the study of Sverrild et al. [56]. This study was randomized, double-blind, placebo-controlled, and conducted as a single-center study. The objective was to identify clinically meaningful changes in the bronchial challenge test with mannitol within 12 weeks of the use of tezepelumab. Further, a secondary outcome would focus on the endobronchial changes of eosinophils, mast cells, and neutrophils expression within the same time frame.

By the end of the monitored period, the tezepelumab group did not show any significant improvement as far as the bronchial challenge was concerned. It was, however, notable that the tezepelumab group did not exert as much airway hyperresponsiveness as the non-tezepelumab group. Regarding the bronchial tissue changes, however, the tezepelumab group showed a significant reduction in eosinophils, as demonstrated in both the bronchial biopsies and the bronchoalveolar lavage cytology. Another interesting and novel finding was a 25% reduction of the mast cells in the tezepelumab group [56].

The decrease in airway eosinophils had also been demonstrated earlier in the study by Gauvreau et al. [57]. This double-blind, placebo-controlled study enrolled 31 mild asthmatics, and no endobronchial biopsies were obtained; still, the anti-TLSP group showed a decreased level in both the peripheral blood and sputum eosinophils despite the allergen challenges performed on days 42 and 84, indicating an amelioration of allergen-induced airway responses and persistent airway inflammation via the anti-TSLP pathway.

As far as the anti-remodeling properties of the anti-TSLP agents, a study by Lin SC et al. [58] has also shown promising results. This experimental study, conducted at the laboratory level on mice, studied the effect of the anti-TSLP agent on matrix metalloproteinase (MMP), connective tissue growth factor (CTGF), and transforming growth factor-β (TGF-β). In ovalbumin-challenged (OVA) mice, the administration of an anti-TSLP mAb decreased airway structural changes by reducing MMP, TGF-β, and connective tissue growth factor, while the thickness of the smooth muscle layer also decreased.

## 7. Discussion

While biologics are gradually becoming better embedded in clinical practice, a detailed understanding of their tissue-level effects, including their impact on airway inflammation and remodeling, remains essential to guide treatment selection and assess therapeutic efficacy. Currently, there is an unmet need in severe asthma, as our biologic choices increase and patients usually present with phenotypes making them eligible for several biologics. Non-invasive biomarkers cannot predict response and/or remission under a certain biologic and furthermore they cannot be used to choose the biologic most likely to achieve the expected outcomes. Finally, few patients demonstrate suboptimal response despite being eligible for a certain biologic, and the reasons behind treatment failure remain unclear. Endobronchial tissue could possibly be the missing piece in the jigsaw of severe asthma to optimize and personalize biologic treatment.

Two recent studies in humans [59,60] demonstrated the existence of two different subpopulations of eosinophils, namely the resident eosinophils (rEos) and the inducible/inflammatory eosinophils (iEos). These eosinophil subtypes are characterized by a different surface molecule expression with a high surface expression of CD62L (CD62Lbright) and low expression of CD101 in rEos, while a low expression of CD62L (CD62Llow) and high expression of CD101 characterized iEos. In the first study by Matucci et al. [59], increased circulating CD62Llow iEos and reduced CD62Lbright rEos were found in patients with severe eosinophilic asthma. Moreover, in the polyp tissue collected from those with severe asthma and concomitant chronic rhinosinusitis with nasal polyps, a higher percentage of CD62Llow iEos was again found. In the other study by Vultaggio et al. [60], treatment with mepolizumab reduced the percentage of CD62Llow iEos in comparison to healthy donor levels.

Mepolizumab seems to interact with iEos, while rEos are mainly involved in tissue homeostasis and are not affected by IL-5 in mice [61]. These studies suggest that mepolizumab and possibly other biologics may exert different effects on subpopulations of eosinophils and such effects at the tissue level may have the potential to enlighten novel insights into the action of a biologic and relate it with success or failure at the clinical level.

In recent years, the fundamental role of the airway epithelium has been acknowledged in severe asthma [62]. Apart from acting as a physical barrier, the airway epithelium possesses many immunological functions that are expressed through the release of various cytokines and especially alarmins that interact with submucosal cells, contributing to both inflammatory and structural changes associated with asthma [63,64].

The alarmins promote eosinophilic inflammation but also induce collagen production by fibroblasts, ASM proliferation and enhanced production of TGF-beta by macrophages [65]. Accordingly, the assessment of the airway epithelium by biopsies taken under bronchoscopy may be a way to precisely recognize the functions of epithelial cells and the respective changes after treatment with a biologic. In such a study, increased production of IL-33 and TSLP was linked to increased asthma severity [66].

Airway remodeling is defined as structural changes of the bronchial wall, both at the epithelium and the submucosa level, such as goblet cell metaplasia, airway smooth muscle cell hyperplasia, subepithelial fibrosis, and angiogenesis [38,67]. Airway remodeling has been associated with more severe asthma, impaired lung function, worse quality of life, and lower response to treatment, so targeting this component of the disease could improve clinical outcomes [38,67].

Some severe asthmatics might develop irreversible airflow obstruction (FEV_1_/FVC < 0.7 and FEV_1_ < 80%), with airway remodeling being the most prevalent factor leading to fixed airway obstruction [68]. This phenotype is thought to occur in 50–60% of patients with severe asthma [69]. Understanding the mechanisms that drive airway remodeling and the effect of biologics to such changes seems essential to enlighten their mechanism of action and also capture the gaps that may lead to the development of new therapies in asthma.

Currently, biomarkers which are used for the monitoring of severe asthma include blood eosinophil count, serum total IgE level, and FeNO level; however, they do not accurately reflect changes at the cellular level. Measurement of airway hyperresponsiveness using provocation tests has also been used to evaluate response to biologic treatment [70]. However, none of the aforementioned markers assesses the epithelium. It is almost definite that the recognition of markers which reflect the integrity and function of the epithelial barrier will enhance the phenotyping and endotyping of severe asthma patients, provided that they could be used in daily clinical practice.

The gold standard for the assessment of airway remodeling remains the obtainment of bronchial biopsies by bronchoscopy. However, bronchoscopy is an invasive technique, particularly in severe asthmatics, and sampling is mainly from the proximal airways and not from the distal small airways which are mainly affected in asthma. For these reasons, it cannot be recommended routinely [69].

The evaluation of response to a biologic and the characterization of a severe asthmatic as non-responder, responder, or super-responder is based on information regarding symptom control, exacerbations, systemic corticosteroid use, and lung function (FEV_1_). The use of biomarkers does not shed light on the changes which take place at the tissue level, ending up in a favorable result. Even more intriguingly, they do not provide information regarding reasons of non-response, which could explain why a patient still experiences symptoms and asthma exacerbations, continues to have lung function impairment, and is unable to cease or decrease the dose of systemic corticosteroids. Such information could be yielded with the use of endobronchial biopsies.

Although bronchoscopy has revealed interesting findings in endobronchial biopsies obtained from severe asthmatics pre- and post-biologic treatment (Table 1), currently there is no documentation of a bronchoscopic/lung tissue predictive factor which would predict the achievement of favorable changes and thus the response to biologic therapy. At the end of the day, the most important clinical decision in the era of personalized medicine is being able to choose the most efficient biologic based on a combination of biomarkers, especially since most patients are eligible for more than one biologic. Such a tissue biomarker could be used alongside other T2 inflammation markers such as blood eosinophils and FeNO, enhancing the strategy in choosing the right biologic and minimizing the risk of treatment failure. Biomarkers for super-responders to a biologic have not been found and this should be essential in studies to be designed. Ideally, the non-responders to a biologic are an intriguing group not only to trace the changes that occur or do not occur at the biopsy level after the administration of a biologic but also to recognize the characteristics at the tissue level of these patients, which might be related with either suboptimal response or treatment failure. This information is also largely lacking. Understanding histologic features in non-responders may inform future biologic development or combination strategies.

A recently published trial evaluated the role of endobronchial biopsies in patients with severe asthma as a predictive factor of response to biologic treatment. This interesting prospective study, which recruited 92 patients from whom 78 managed to complete it, assigned patients to either anti-IL-5 (mepolizumab, benralizumab or reslizumab) or anti-IL-4/13 (dupilumab) treatment. A total of 63 patients were under anti-IL-5 treatment and the remaining 15 were under dupilumab. Bronchial biopsies were evaluated in terms of submucosal inflammation and presence of eosinophils, epithelial membrane changes, thickening of basement membrane, and submucosal mucus glands. From these five parameters, tissue eosinophilia was found to be the most decisive predictive factor. This five-item score (PS score) was compared to a T2 score, which was based on peripheral blood eosinophils and FeNO levels. The PS score was the only variable independently associated with response and super-response to biologic treatment. Moreover, the PS score was superior compared to the T2 score in predicting treatment response in patients under OCS treatment and in those who received anti-IL-5 therapy. These findings pave the way for routine endobronchial biopsies in severe asthmatics, especially in those who do not initially respond to treatment in an effort for a better phenotyping of these patients [71]. The next steps should be taken in the path of using bronchial biopsies to select the right biologic for each patient.

Mucus plugging has been found to be present in a large proportion of patients with severe asthma and is a constant feature, persisting for at least 3 years [72,73]. Baseline mucus plugging scores have been correlated positively with blood eosinophil count, eosinophil-derived neurotoxin, FeNO, IL-5, and IL-13, and negatively with lung function (FEV_1_ and FEF_25–75%_) years [73]. Biologic therapies such as anti-IL-5/5R and anti-TSLP have been shown to reduce mucus plugs and this reduction has been associated with improvements in lung function and asthma symptoms [55,74]

High-resolution computed tomography (HRCT) has been used in some studies with biologics as a non-invasive tool for the assessment of airway remodeling. Despite the progress in these techniques and the fact that there are ongoing relevant studies such as FUNLUM for mepolizumab and BURAN for benralizumab that will assess with functional respiratory imaging (FRI) remodeling changes, such studies lack the ability to enlighten mechanisms at the tissue level, which lead to the radiographic finding [75].

With the incorporation of the “remission” notion in current severe asthma management, earlier intervention with biologics blocking initial events may be useful in preventing asthma or the progression of milder to more severe disease. Accordingly, the use of blood eosinophil count and FeNO along with novel biomarkers reflecting the tissue in severe asthma could play a critical role in optimizing the use of biologics in preventing disease progression and intervene in disease modification.

## Figures and Tables

**Table 1:** Studies of biologics and findings from biopsies in severe asthma.

Omalizumab				
Author (Year)	Type of Study	Study Sample	Focus on Biopsies	Main Findings
Djukanović et al. (2004) [24]	Prospective, randomized controlled trial	45 patients (22 omalizumab, 23 placebo), mild to moderate persistent asthma with ≥2% sputum eosinophils	Bronchial biopsies at baseline and post-treatment	↓ eosinophils, IgE+ cells, CD3^+^, CD4^+^, CD8^+^ T-cells, B-cells, and IL-4^+^ cells in omalizumab group ~ airway hyperresponsiveness
van Rensen et al. (2009) [25]	Double-blind, randomized, placebo-controlled trial	25 participants with asthma	Bronchial biopsies 24h after allergen challenge and after 12 weeks of treatment	↓ airway eosinophils, CD4^+^ T cells, and FcεRI^+^ cells in omalizumab group ~ in airway hyperresponsiveness (PC20)
Riccio et al. (2012) [26]	Prospective, non-randomized	11 patients with severe persistent allergic asthma	Bronchial biopsies before and after treatment	↓ RBM thickness and eosinophils in the omalizumab group
Mauri et al. (2014) [27]	Prospective, non-randomized study	8 patients with severe persistent atopic asthma	Bronchial biopsies pre- and post-treatment (12 months)	↓ smooth muscle-associated proteins (myosins, actins) in the omalizumab group ~ in ECM proteins; galectin-3 found only in responders
Pillai et al. (2016) [28]	Prospective, randomized clinical trial	18 patients with non-atopic asthma (9 on omalizumab, 9 on placebo) over 20 weeks	Bronchial biopsies obtained at baseline and after 12–14 weeks	↓ IgE-expressing cells in the omalizumab preserved or ↑ FEV_1_ in omalizumab (Placebo group showed decline in lung function)
Huang et al. (2019) [29]	Prospective, non-randomized study	23 patients with severe allergic asthma; 14 responders	Bronchial biopsies before and after omalizumab treatment	↓ IL-33, IL-25, and TSLP observed in responders. Clinical improvements in exacerbations, asthma control, and lung function
Zastrzeżyńska et al. (2020) [31]	Prospective, non-randomized	13 patients with severe allergic asthma	Bronchial biopsies at baseline and after 12 months	↓ basal lamina thickness and fibronectin; ~ collagen
**Mepolizumab**				
**Author (Year)**	**Type of Study**	**Study Sample**	**Focus on Biopsies**	**Main Findings**
Kelly et al. (2017) [36]	Experimental, non-clinical prospective study	38 mild asthmatics enrolled, 10 completed the study	WLAC → 1 month bronchoscopy at 0 and 48 h following SBP-Ag → 1 month 750 mg mepolizumab → 4–12 weeks bronchoscopy at 0 and 48 h following SBP-Ag post mepolizumab	↓ airway eosinophils ↓ eosinophilic peroxidase deposition Remaining eosinophils express functional cytokine and IL-3 and IL-5 receptors in response to allergen challenge
Domvri et al. (2025) [37]	Prospective non-randomized multicenter study	41 severe T2 high asthmatics, 34 had pre and post mepolizumab bronchial biopsies	Bronchial biopsies at baseline and after 12 months	↓ sub-basement membrane thickness ↓ airway smooth muscle area/layer thickness ↓ epithelial damage ↓ tissue eosinophils
Flood-Page et al. (2003) [39]	Double blind placebo-controlled trial	24 mild asthmatics	Bronchial biopsies at baseline and after 3 months	↓ tissue eosinophils ↓ TGF-β levels ↓ extracellular matrix proteins (e.g., tenascine)
Taille et al. (2024) [40]	Single center prospective non-randomized study	37 severe asthmatics 23 completed the study	Bronchial biopsies at baseline and after 6 and 12 months	↓ sub-basement membrane thickness ↓ smooth muscle cell area ↓ tissue eosinophils at 6 months, persisting through 12 months
**Benralizumab**				
**Author (Year)**	**Type of Study**	**Study Sample**	**Focus on Biopsies**	**Main Findings**
Laviolette et al. (2013) [44]	Prospective double-blind placebo-controlled study	13 patients single IV shot or placebo 14 patients SC 100 mg, 200 mg or placebo Q4W	Bronchial biopsies at baseline and after 3 months	↓ airway and sputum eosinophils Complete peripheral blood eosinophil abolishment SC inferior to IV regimen
Chachi et al. (2019) [45]	Retrospective study	15 subjects receiving benralizumab and 10 subjects receiving placebo from the trial of Laviolette et al. (2013)	Bronchial biopsies at baseline and after 3 months	↓ airway smooth muscle mass by 29% in the benralizumab group pre-and post-treatment No significant difference between treatment and placebo group post-treatment
Gauvreau et al. (2024) [46]	Prospective randomized placebo-controlled study	46 mild asthmatics 30 mg dose Q4W for 3 months	Bronchial biopsies at baseline and after 3 months post-allergen challenge	↓ tissue, sputum, and blood eosinophils Allergen induced bronchoconstriction persists despite eosinophil depletion
NCT03953300 (ongoing) [47]	Phase IV multicenter randomized double blind, parallel group, placebo-controlled trial	Severe asthmatic patients under anti-IL-5R treatment	Bronchial biopsies—Structural lung changes	ETA by the end of 2026
NCT04365205 (ongoing) [48]	Prospective observational study	60 patients, 20 severe asthmatics, 20 mild-moderate asthmatics, and 20 non-asthmatics	Bronchial biopsies and BSM cell culture In vitro assessment of airway remodeling	Original ETA by the end of 2024 but patients are still being enrolled
**Dupilumab**				
**Author (Year)**	**Type of Study**	**Study Sample**	**Focus on Biopsies**	**Main Findings**
NCT02573233 (EXPEDITION) [50]	Phase IIa exploratory trial	42 patients 1:1 randomization	Bronchial biopsies at baseline and after 12 weeks	Eosinophil and mast cell levels not reduced ↓ mucin-stained submucosal area ↓ FeNO
Bini et al. (2023) [51]	Single center prospective study	10 patients under dupilumab	Bronchial biopsies at baseline and after 6 months	Eosinophils depleted in BAL—persist in bronchial mucosa
**Tezepelumab**				
**Author (Year)**	**Type of Study**	**Study Sample**	**Focus on Biopsies**	**Main Findings**
Diver et al. “CASCADE” (2021) [55]	Phase 2, randomized, double-blind, placebo-controlled trial	116 patients (59 tezepelumab, 57 placebo)	Bronchoscopic biopsies pre- and post-treatment	89% ↓ in submucosal eosinophils vs. 25% with placebo ~ in other inflammatory cells ~ in RBM or epithelial morphology
Sverrild et al. (2021) [56]	Randomized, double-blind, placebo-controlled, single-center study	Patients with moderate to severe asthma; 12-week treatment period with Tezepelumab	Bronchial biopsies and BAL	↓ airway hyperresponsiveness 74% ↓in bronchial eosinophils 25% ↓ in bronchial mast cells
Gauvreau et al. (2014) [57]	Double-blind, placebo-controlled study	31 patients with mild asthma	No endobronchial biopsy performed	↓ blood and sputum eosinophils in the anti-TSLP group, despite allergen challenges on days 42 and 84
Lin et al. (2019) [58]	Experimental (in vivo, mice model)	OVA-challenged mice (animal model of asthma)	No bronchial biopsy; assessment via tissue analysis post-sacrifice	↓ airway structural changes ↓MMP, CTGF, and TGF-β) ↓ smooth muscle layer thickness

Abbreviations: ↓: reduction, ~: no or minimal effect, ↑: increase, IL-4: interleukin-4, PC20: Provocative Concentration 20, RBM: Reticular basement membrane, ECM: Extracellular cell matrix, FEV_1_: Forced expiratory volume in the 1^st^ second, IL-33: interleukin-33, IL-25: interleukin-25, TSLP: Thymic Stromal Lymphopoietin, WLAC: Whole lung allergen challenge, SBP-Ag: Segmental Bronchial Provocation with allergen, IL-3: interleukin-3, IL-5: interleukin-5, TGF-β: Transforming growth factor beta, Q4W: every 4 weeks, IV: intravenous, SC: subcutaneous, IL-5R: interleukin-5 receptor, ETA: estimated time of arrival, BSM: Bronchial Smooth Muscle, FeNO: Fractional Exhaled Nitric Oxide, BAL: Bronchoalveolar Lavage, OVA: Ovalbumin MMP: Matrix Metalloproteinases, CTGF: Connective Tissue Growth Factor.
